# Bis(4-amino­pyridinium) hexa­aqua­nickel(II) bis­(sulfate)

**DOI:** 10.1107/S1600536813032558

**Published:** 2013-12-07

**Authors:** Thameur Sahbani, Wajda Smirani Sta, Mohamed Rzaigui

**Affiliations:** a Laboratoire de Chimie des Matériaux, Faculté des Sciences de Bizerte, 7021 Zarzouna Bizerte, Tunisia

## Abstract

In the title compound, (C_5_H_7_N_2_)_2_[Ni(H_2_O)_6_](SO_4_)_2_, the Ni^II^ cation is located on an inversion centre and is coordinated by six aqua ligands in a slightly distorted octa­hedral coordination environment. The [Ni(H_2_O)_6_]^2+^ ions are connected through an extensive network of O—H⋯O hydrogen bonds to sulfate anions, leading to the formation of layers parallel to (001). The 4-amino­pyridinium cations are located between these layers and are connected to the anionic framework by N—H⋯O hydrogen bonds. Weak π–π inter­actions between the pyridine rings, with a centroid–centroid distance of 3.754 (9) Å, provide additional stability to the crystal packing.

## Related literature   

For applications of metal sulfate complexes, see: Rekik *et al.* (2008 ▶). For clinical background to 4-amino­pyridine, see: Judge & Bever (2006 ▶); Schwid *et al.* (1997 ▶); Strupp *et al.* (2004 ▶). For related compounds, see: Anderson *et al.* (2005 ▶); Hajlaoui *et al.* (2011 ▶); Quah *et al.* (2010 ▶); Rotondo *et al.* (2009 ▶).
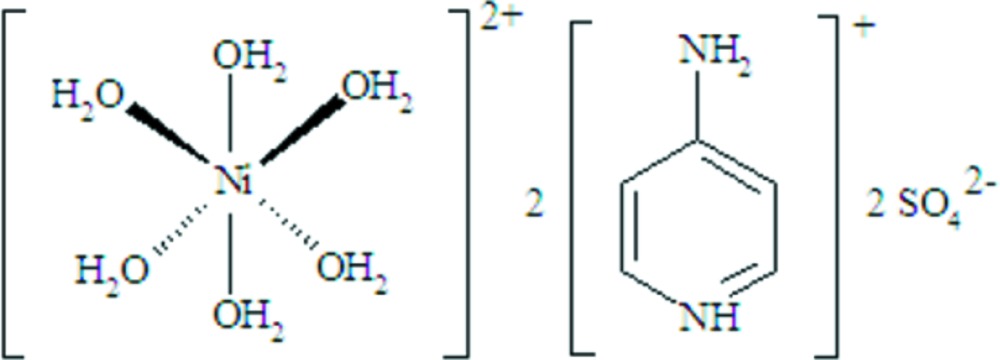


## Experimental   

### 

#### Crystal data  

(C_5_H_7_N_2_)_2_[Ni(H_2_O)_6_](SO_4_)_2_
*M* *_r_* = 549.18
Triclinic, 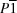

*a* = 6.212 (2) Å
*b* = 7.015 (3) Å
*c* = 12.422 (2) Å
α = 100.61 (3)°
β = 99.26 (2)°
γ = 99.57 (2)°
*V* = 514.3 (3) Å^3^
*Z* = 1
Ag *K*α radiation
λ = 0.56085 Å
μ = 0.64 mm^−1^
*T* = 293 K
0.30 × 0.20 × 0.20 mm

#### Data collection  

Enraf–Nonius TurboCAD-4 diffractometer
5919 measured reflections
5022 independent reflections
3986 reflections with *I* > 2σ(*I*)
*R*_int_ = 0.014
2 standard reflections every 120 min intensity decay: 5%

#### Refinement  

*R*[*F* ^2^ > 2σ(*F* ^2^)] = 0.038
*wR*(*F* ^2^) = 0.097
*S* = 1.11
5022 reflections
174 parameters
6 restraints
H atoms treated by a mixture of independent and constrained refinement
Δρ_max_ = 0.75 e Å^−3^
Δρ_min_ = −1.06 e Å^−3^

### 

Data collection: *CAD-4 EXPRESS* (Enraf–Nonius, 1994 ▶); cell refinement: *CAD-4 EXPRESS*; data reduction: *XCAD4* (Harms & Wocadlo, 1995 ▶); program(s) used to solve structure: *SHELXS97* (Sheldrick, 2008 ▶); program(s) used to refine structure: *SHELXL97* (Sheldrick, 2008 ▶); molecular graphics: *ORTEP-3 for Windows* (Farrugia, 2012 ▶); software used to prepare material for publication: *WinGX* (Farrugia, 2012 ▶).

## Supplementary Material

Crystal structure: contains datablock(s) I, cad4. DOI: 10.1107/S1600536813032558/wm2787sup1.cif

Structure factors: contains datablock(s) I. DOI: 10.1107/S1600536813032558/wm2787Isup2.hkl

Additional supporting information:  crystallographic information; 3D view; checkCIF report

## Figures and Tables

**Table 1:** Hydrogen-bond geometry (Å, °)

*D*—H⋯*A*	*D*—H	H⋯*A*	*D*⋯*A*	*D*—H⋯*A*
N2—H1⋯O2^i^	0.86	2.43	3.102 (2)	136
N2—H1⋯O3^ii^	0.86	2.23	2.983 (2)	147
O1—H1*O*1⋯O7^i^	0.82 (2)	2.03 (2)	2.8231 (16)	163 (2)
O1—H2*O*1⋯O5^iii^	0.86 (2)	1.82 (2)	2.6741 (16)	172 (2)
O2—H1*O*2⋯O4^iv^	0.83 (2)	1.90 (2)	2.6952 (17)	161 (2)
O2—H2*O*2⋯O7^i^	0.84 (2)	1.95 (2)	2.7480 (16)	159 (2)
N1—H6⋯O4^i^	0.80 (3)	2.17 (3)	2.957 (2)	173 (3)
N1—H7⋯O5^v^	0.86 (3)	2.55 (3)	3.153 (2)	129 (2)
N1—H7⋯O6^v^	0.86 (3)	2.15 (3)	2.998 (2)	170 (2)
O3—H103⋯O5^vi^	0.77 (2)	1.96 (2)	2.7072 (15)	164 (2)
O3—H203⋯O7^vii^	0.815 (19)	1.876 (19)	2.6682 (15)	164 (2)

Symmetry codes: (i) 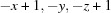; (ii) 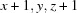; (iii) 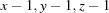; (iv) 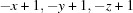; (v) 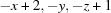; (vi) 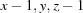; (vii) 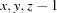.

**Fig. 1.**
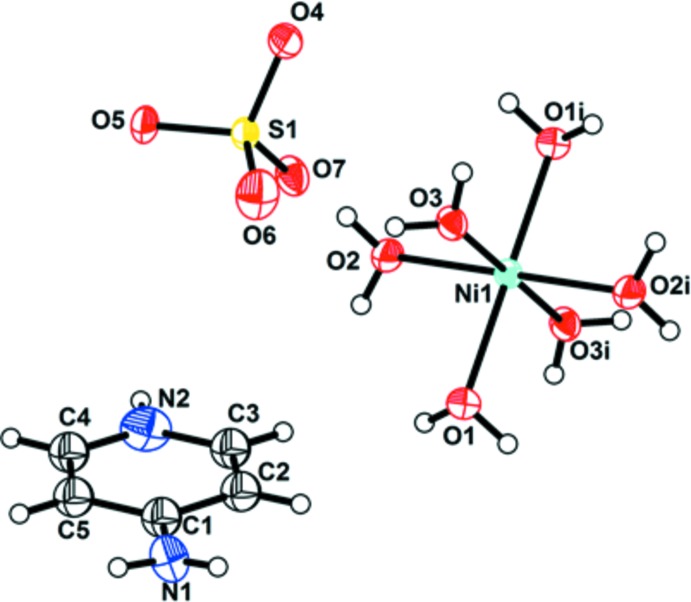
View of the molecular entities of the title compound with atom numbering scheme. Displacement ellipsoids for non-H atoms are drawn at the 30% probability level. [Symmetry code: i) -x, -y, -z].

**Fig. 2.**
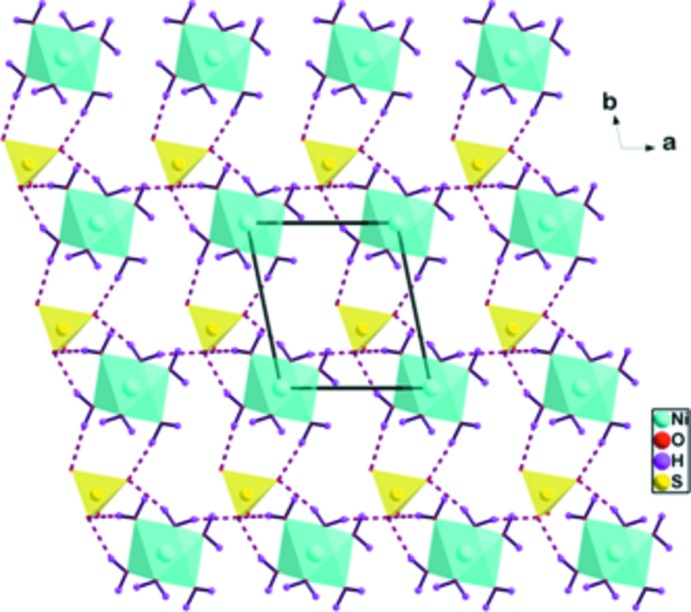
View of a corrugated layer of the title compound parallel to (001). Sulfate polyhedra are shown in yellow and cobalt polyhadra in cyan. Hydrogen bonds are denoted by dashed lines.

**Fig. 3.**
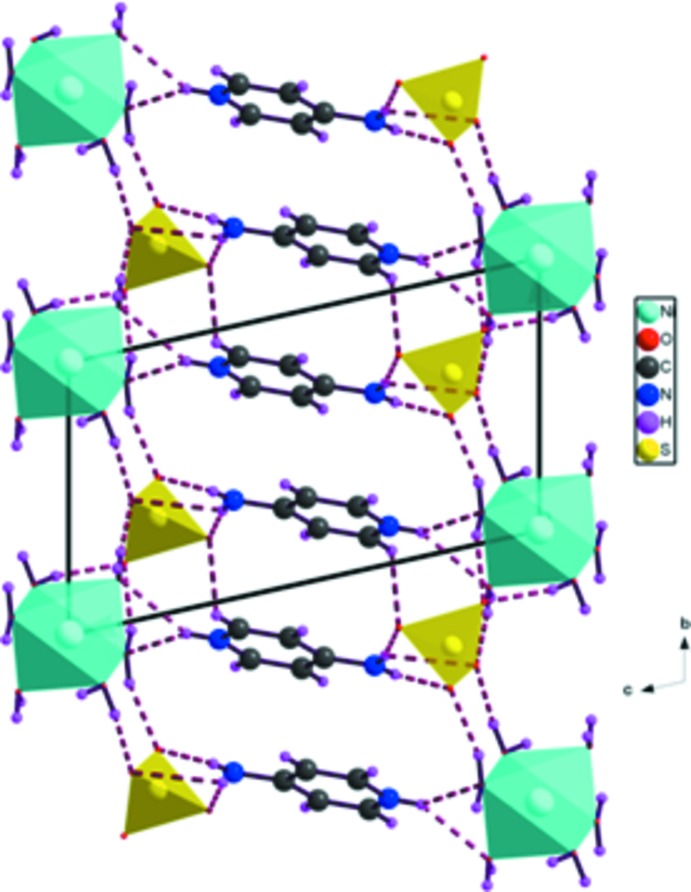
View of the molecular arrangement in the title structure viewed along [100]. Hydrogen bonds are denoted by dashed lines.

## supplementary crystallographic information

### 1. Comment

4-Aminopyridine (fampridine) is clinically used in the treatment of the Lambert-Eaton myasthenic syndrome and of multiple sclerosis. It prolongs action potentials by blocking potassium channels, thereby increases transmitter release at the neuromuscular junction (Judge & Bever, 2006; Schwid *et al.*, 1997; Strupp *et al.*, 2004). The combination of this amine with sulfuric acid leads to the formation of bis(4-aminopyridinium) sulfate monohydrate (Quah *et al.*, 2010). In general, sulfates combined with transition metal and organic groups can lead to interesting materials as model compounds of ferroelectric and ferroelastic domains, but also to potentially applicable compounds with interesting magnetic properties (Rekik *et al.*, 2008). For this reasons, we focused to study the crystal structure of the title compound, (C_5_H_7_N_2_)_2_[Ni(H_2_O)_6_](SO_4_)_2_, (I).

The crystal structure of compound (I) has an asymmetric unit consisting of one half of the cationic complex [Ni(H_2_O)_6_]^2+^, an uncoordinating sulfate anion, and one 4-aminopyridinium cation, C_5_H_7_N_2_^2+^ (Fig. 1). The Ni(II) ion, located on an inversion centre, exhibits a distorted octahedral coordination environment with Ni–O distances ranging from 2.0388 (11) to 2.0701 (12) Å. The values of O—Ni—O angles are between 86.04 (5) and 180.00 (7). These values agree with those for other [Ni(H_2_O)_6_]^2+^ groups (Hajlaoui *et al.*, 2011).

A proton transfer from sulfuric acid to atom N2 of 4-aminopyridine resulted in the formation of a 4-aminopyridinium cation. This protonation leads to the widening of the C3–N2–C4 angle of the pyridine ring to 121.02 (15)°, compared to 115.25 (13)° in the unprotonated 4-aminopyridine (Anderson *et al.*, 2005). Such a protonation is observed in various 4-aminopyridine acid complexes (Rotondo *et al.*, 2009). The 4-aminopyridine ring is essentially planar with a maximum deviation from planarity of 0.002 (9) Å.

In the crystal structure, intermolecular O—H···O hydrogen bonds, established between the complex cations and sulfate anions, generate layers parallel to (001) (Fig. 2). The crystal packing of the title complex is stabilized by N—H···O hydrogen bonds between C_5_H_7_N_2_^2+^ cations and sulfate anions and by *π*···*π* interactions between parallel pyridine rings [ring centroid-to-centroid distance: 3.754 (9) Å], which link the different species into a three dimensional network (Fig. 3).

### 2. Experimental

4-Aminopyridine (0.19g, 2 mmol) and nickel sulfate hexahydrate (0.26g, 1 mmol) were dissolved in 15 ml of water. The resulting solution was added to an aqueous solution of sulfuric acid (0.1 M). The mixture was stirred for 20 min at room temperature. After slow evaporation during few days at ambient temperature, blue single crystals of the title compound appeared in the solution.

### 3. Refinement

The H atoms bonded to C and N2 atoms were positioned geometrically and treated as riding on their parent atoms, [N—H = 0.86, C—H =0.93 Å with *U*_iso_(H) = 1.2 *U*_eq_(C,N), but those attached to N1 were located in a difference Fourier map and refined freely. O—H bond lengths and distances between two H atoms of each water molecule were restrained to be 0.85 (2) and 1.37 (2) Å, with *U*_iso_(H) = 1.5 *U*_eq_(O).

### Crystal data

**Table:**

(C_5_H_7_N_2_)_2_[Ni(H_2_O)_6_](SO_4_)_2_	*Z* = 1
*M**_r_* = 549.18	*F*(000) = 286
Triclinic, *P*1	*D*_x_ = 1.773 Mg m^−^^3^
Hall symbol: -P 1	Ag *K*α radiation, λ = 0.56085 Å
*a* = 6.212 (2) Å	Cell parameters from 25 reflections
*b* = 7.015 (3) Å	θ = 9–11°
*c* = 12.422 (2) Å	µ = 0.64 mm^−^^1^
α = 100.61 (3)°	*T* = 293 K
β = 99.26 (2)°	Block, blue
γ = 99.57 (2)°	0.30 × 0.20 × 0.20 mm
*V* = 514.3 (3) Å^3^	

### Data collection

**Table:**

Enraf–Nonius TurboCAD-4 diffractometer	*R*_int_ = 0.014
Radiation source: fine-focus sealed tube	θ_max_ = 28.0°, θ_min_ = 2.4°
Graphite monochromator	*h* = −10→10
ω scans	*k* = −11→11
5919 measured reflections	*l* = −2→20
5022 independent reflections	2 standard reflections every 120 min
3986 reflections with *I* > 2σ(*I*)	intensity decay: 5%

### Refinement

**Table:**

Refinement on *F*^2^	Primary atom site location: structure-invariant direct methods
Least-squares matrix: full	Secondary atom site location: difference Fourier map
*R*[*F*^2^ > 2σ(*F*^2^)] = 0.038	Hydrogen site location: inferred from neighbouring sites
*wR*(*F*^2^) = 0.097	H atoms treated by a mixture of independent and constrained refinement
*S* = 1.11	*w* = 1/[σ^2^(*F*_o_^2^) + (0.0459*P*)^2^ + 0.2397*P*] where *P* = (*F*_o_^2^ + 2*F*_c_^2^)/3
5022 reflections	(Δ/σ)_max_ = 0.001
174 parameters	Δρ_max_ = 0.75 e Å^−^^3^
6 restraints	Δρ_min_ = −1.06 e Å^−^^3^

### Special details

**Table:**

Geometry. All esds (except the esd in the dihedral angle between two l.s. planes) are estimated using the full covariance matrix. The cell esds are taken into account individually in the estimation of esds in distances, angles and torsion angles; correlations between esds in cell parameters are only used when they are defined by crystal symmetry. An approximate (isotropic) treatment of cell esds is used for estimating esds involving l.s. planes.
Refinement. Refinement of F^2^ against ALL reflections. The weighted R-factor wR and goodness of fit S are based on F^2^, conventional R-factors R are based on F, with F set to zero for negative F^2^. The threshold expression of F^2^ > 2sigma(F^2^) is used only for calculating R-factors(gt) etc. and is not relevant to the choice of reflections for refinement. R-factors based on F^2^ are statistically about twice as large as those based on F, and R- factors based on ALL data will be even larger.

### Fractional atomic coordinates and isotropic or equivalent isotropic displacement parameters (Å^2^)

**Table:**

	*x*	*y*	*z*	*U*_iso_*/*U*_eq_	
Ni1	0.0000	0.0000	0.0000	0.01654 (6)	
O1	0.15341 (17)	−0.21342 (15)	−0.07350 (9)	0.02370 (18)	
O2	0.28416 (17)	0.11424 (15)	0.12233 (10)	0.0257 (2)	
O3	0.10291 (17)	0.16756 (15)	−0.10704 (9)	0.02297 (18)	
C1	0.9486 (2)	−0.2906 (2)	0.45752 (12)	0.0243 (2)	
N2	0.9660 (3)	−0.1384 (2)	0.68016 (12)	0.0418 (4)	
H1	0.9714	−0.0903	0.7496	0.050*	
C3	0.7706 (3)	−0.2354 (3)	0.61472 (15)	0.0395 (4)	
H3	0.6434	−0.2507	0.6454	0.047*	
C5	1.1512 (3)	−0.1901 (2)	0.53005 (15)	0.0316 (3)	
H5	1.2830	−0.1752	0.5032	0.038*	
C4	1.1524 (3)	−0.1156 (3)	0.63905 (16)	0.0389 (4)	
H4	1.2856	−0.0473	0.6861	0.047*	
N1	0.9396 (3)	−0.3609 (3)	0.35006 (12)	0.0338 (3)	
C2	0.7559 (3)	−0.3115 (3)	0.50454 (13)	0.0305 (3)	
H2	0.6191	−0.3773	0.4601	0.037*	
H103	0.056 (4)	0.261 (3)	−0.1106 (18)	0.030 (5)*	
H203	0.236 (3)	0.205 (3)	−0.1029 (16)	0.023 (4)*	
H7	1.061 (5)	−0.333 (4)	0.327 (2)	0.062 (8)*	
H6	0.827 (5)	−0.410 (4)	0.307 (2)	0.048 (7)*	
S1	0.62063 (5)	0.35433 (4)	0.81367 (3)	0.02033 (7)	
O5	0.85539 (16)	0.44487 (16)	0.86606 (11)	0.0306 (2)	
O7	0.53371 (17)	0.21768 (17)	0.88249 (10)	0.0295 (2)	
O4	0.48753 (19)	0.50858 (17)	0.81131 (13)	0.0354 (3)	
O6	0.6085 (2)	0.2407 (2)	0.70136 (11)	0.0420 (3)	
H1O1	0.257 (3)	−0.228 (3)	−0.0278 (18)	0.047 (7)*	
H2O1	0.065 (4)	−0.327 (3)	−0.098 (2)	0.059 (8)*	
H1O2	0.363 (4)	0.225 (2)	0.129 (2)	0.047 (7)*	
H2O2	0.367 (3)	0.034 (3)	0.1328 (19)	0.032 (5)*	

### Atomic displacement parameters (Å^2^)

**Table:**

	*U*^11^	*U*^22^	*U*^33^	*U*^12^	*U*^13^	*U*^23^
Ni1	0.01578 (10)	0.01730 (10)	0.01684 (10)	0.00301 (7)	0.00362 (7)	0.00442 (7)
O1	0.0239 (4)	0.0213 (4)	0.0263 (5)	0.0059 (3)	0.0053 (4)	0.0045 (4)
O2	0.0219 (4)	0.0216 (4)	0.0301 (5)	0.0031 (3)	−0.0012 (4)	0.0038 (4)
O3	0.0195 (4)	0.0242 (4)	0.0287 (5)	0.0051 (3)	0.0078 (4)	0.0113 (4)
C1	0.0253 (6)	0.0268 (6)	0.0222 (6)	0.0068 (5)	0.0044 (5)	0.0081 (5)
N2	0.0545 (10)	0.0452 (8)	0.0222 (6)	0.0137 (7)	0.0035 (6)	−0.0014 (6)
C3	0.0401 (9)	0.0538 (10)	0.0278 (7)	0.0136 (8)	0.0131 (7)	0.0080 (7)
C5	0.0255 (6)	0.0327 (7)	0.0340 (7)	0.0020 (5)	0.0014 (6)	0.0079 (6)
C4	0.0395 (8)	0.0317 (8)	0.0358 (8)	0.0025 (6)	−0.0064 (7)	−0.0006 (6)
N1	0.0303 (6)	0.0498 (8)	0.0217 (6)	0.0093 (6)	0.0061 (5)	0.0071 (6)
C2	0.0256 (6)	0.0398 (8)	0.0253 (6)	0.0043 (6)	0.0061 (5)	0.0064 (6)
S1	0.01526 (12)	0.02018 (13)	0.02486 (15)	0.00201 (10)	0.00201 (10)	0.00623 (11)
O5	0.0164 (4)	0.0257 (5)	0.0454 (7)	−0.0001 (3)	−0.0019 (4)	0.0076 (5)
O7	0.0222 (4)	0.0331 (5)	0.0390 (6)	0.0067 (4)	0.0098 (4)	0.0184 (5)
O4	0.0231 (5)	0.0246 (5)	0.0571 (8)	0.0068 (4)	−0.0024 (5)	0.0124 (5)
O6	0.0400 (7)	0.0518 (8)	0.0275 (6)	0.0023 (6)	0.0070 (5)	−0.0019 (5)

### Geometric parameters (Å, º)

**Table:**

Ni1—O3^i^	2.0388 (11)	N2—C4	1.336 (3)
Ni1—O3	2.0388 (11)	N2—C3	1.342 (3)
Ni1—O1	2.0578 (12)	N2—H1	0.8600
Ni1—O1^i^	2.0578 (12)	C3—C2	1.357 (2)
Ni1—O2^i^	2.0701 (12)	C3—H3	0.9300
Ni1—O2	2.0701 (12)	C5—C4	1.357 (3)
O1—H1O1	0.818 (15)	C5—H5	0.9300
O1—H2O1	0.860 (15)	C4—H4	0.9300
O2—H1O2	0.828 (15)	N1—H7	0.85 (3)
O2—H2O2	0.839 (14)	N1—H6	0.79 (3)
O3—H103	0.77 (2)	C2—H2	0.9300
O3—H203	0.82 (2)	S1—O6	1.4574 (14)
C1—N1	1.324 (2)	S1—O4	1.4686 (12)
C1—C2	1.412 (2)	S1—O5	1.4793 (12)
C1—C5	1.413 (2)	S1—O7	1.4897 (12)
			
O3^i^—Ni1—O3	180.00 (5)	C2—C1—C5	117.08 (14)
O3^i^—Ni1—O1	92.26 (5)	C4—N2—C3	121.02 (15)
O3—Ni1—O1	87.74 (5)	C4—N2—H1	119.5
O3^i^—Ni1—O1^i^	87.74 (5)	C3—N2—H1	119.5
O3—Ni1—O1^i^	92.26 (5)	N2—C3—C2	121.01 (17)
O1—Ni1—O1^i^	180.00 (7)	N2—C3—H3	119.5
O3^i^—Ni1—O2^i^	93.96 (5)	C2—C3—H3	119.5
O3—Ni1—O2^i^	86.04 (5)	C4—C5—C1	119.69 (16)
O1—Ni1—O2^i^	89.83 (5)	C4—C5—H5	120.2
O1^i^—Ni1—O2^i^	90.17 (5)	C1—C5—H5	120.2
O3^i^—Ni1—O2	86.04 (5)	N2—C4—C5	121.31 (16)
O3—Ni1—O2	93.96 (5)	N2—C4—H4	119.3
O1—Ni1—O2	90.17 (5)	C5—C4—H4	119.3
O1^i^—Ni1—O2	89.83 (5)	C1—N1—H7	115.4 (19)
O2^i^—Ni1—O2	180.00 (7)	C1—N1—H6	123 (2)
Ni1—O1—H1O1	109.7 (18)	H7—N1—H6	121 (3)
Ni1—O1—H2O1	112.7 (19)	C3—C2—C1	119.88 (16)
H1O1—O1—H2O1	107.0 (19)	C3—C2—H2	120.1
Ni1—O2—H1O2	123.5 (17)	C1—C2—H2	120.1
Ni1—O2—H2O2	114.9 (14)	O6—S1—O4	111.02 (9)
H1O2—O2—H2O2	108.4 (18)	O6—S1—O5	109.76 (8)
Ni1—O3—H103	118.3 (16)	O4—S1—O5	110.07 (7)
Ni1—O3—H203	118.9 (13)	O6—S1—O7	108.89 (8)
H103—O3—H203	103.5 (18)	O4—S1—O7	109.07 (8)
N1—C1—C2	121.41 (14)	O5—S1—O7	107.96 (7)
N1—C1—C5	121.51 (14)		

Symmetry code: (i) −*x*, −*y*, −*z*.

### Hydrogen-bond geometry (Å, º)

**Table:**

*D*—H···*A*	*D*—H	H···*A*	*D*···*A*	*D*—H···*A*
N2—H1···O2^ii^	0.86	2.43	3.102 (2)	136
N2—H1···O3^iii^	0.86	2.23	2.983 (2)	147
O1—H1*O*1···O7^ii^	0.82 (2)	2.03 (2)	2.8231 (16)	163 (2)
O1—H2*O*1···O5^iv^	0.86 (2)	1.82 (2)	2.6741 (16)	172 (2)
O2—H1*O*2···O4^v^	0.83 (2)	1.90 (2)	2.6952 (17)	161 (2)
O2—H2*O*2···O7^ii^	0.84 (2)	1.95 (2)	2.7480 (16)	159 (2)
N1—H6···O4^ii^	0.80 (3)	2.17 (3)	2.957 (2)	173 (3)
N1—H7···O5^vi^	0.86 (3)	2.55 (3)	3.153 (2)	129 (2)
N1—H7···O6^vi^	0.86 (3)	2.15 (3)	2.998 (2)	170 (2)
O3—H103···O5^vii^	0.77 (2)	1.96 (2)	2.7072 (15)	164 (2)
O3—H203···O7^viii^	0.815 (19)	1.876 (19)	2.6682 (15)	164 (2)

Symmetry codes: (ii) −*x*+1, −*y*, −*z*+1; (iii) *x*+1, *y*, *z*+1; (iv) *x*−1, *y*−1, *z*−1; (v) −*x*+1, −*y*+1, −*z*+1; (vi) −*x*+2, −*y*, −*z*+1; (vii) *x*−1, *y*, *z*−1; (viii) *x*, *y*, *z*−1.
